# Self-reported infections during international travel and notifiable infections among returning international travellers, Sweden, 2009-2013

**DOI:** 10.1371/journal.pone.0181625

**Published:** 2017-07-28

**Authors:** Viktor Dahl, Anders Wallensten

**Affiliations:** 1 The Public Health Agency of Sweden, Stockholm, Sweden; 2 European Programme for Intervention Epidemiology Training (EPIET), European Centre for Disease Prevention and Control (ECDC), Stockholm, Sweden; 3 Department of Medical Sciences, Uppsala University, Uppsala, Sweden; Umeå University, SWEDEN

## Abstract

We studied food and water-borne diseases (FWDs), sexually transmitted diseases (STDs), vector-borne diseases (VBDs) and diseases vaccinated against in the Swedish childhood vaccination programme among Swedish international travellers, in order to identify countries associated with a high number of infections. We used the national database for notifiable infections to estimate the number of FWDs (campylobacteriosis, salmonellosis, giardiasis, shigellosis, EHEC, Entamoeba histolytica, yersinosis, hepatitis A, paratyphoid fever, typhoid fever, hepatitis E, listeriosis, cholera), STIs (chlamydia, gonorrhoea and acute hepatitis B), VBDs (dengue fever, malaria, West Nile fever, Japanese encephalitis and yellow fever) and diseases vaccinated against in the Swedish childhood vaccination programme (pertussis, measles, mumps, rubella, diphtheria) acquired abroad 2009–2013. We obtained number and duration of trips to each country from a database that monthly collects travel data from a randomly selected proportion of the Swedish population. We calculated number of infections per country 2009–2013 and incidence/million travel days for the five countries with the highest number of infections. Thailand had the highest number of FWDs (7,697, incidence 191/million travel days), STIs (1,388, incidence 34/million travel days) and VBDs (358, incidence 9/million travel days). France had the highest number of cases of diseases vaccinated against in the Swedish childhood vaccination programme (8, 0.4/million travel days). Swedish travellers contracted most infections in Thailand. Special focus should be placed on giving advice to travellers to this destination.

## Introduction

In 2014 the annual number of international overnight tourists exceeded 1,133 million globally and is estimated to reach 1,800 million by 2030 [1]. International travellers are at risk of contracting infections while abroad. The disease panorama and the risk for infection may be different at their destinations compared to in their country of origin. For example, In 2010–2012, the most common health problem among Finnish international travellers seeking medical care abroad was infections [2]. Finland is a neighbouring country to Sweden and is in many ways similar to Sweden.

Studies on travel-associated infections from both the US and Europe are often limited by a lack of denominator data (i.e. number of trips to and/or days spent in each country) which prevents incidence calculation [3–6]. Since 1989, in Sweden, a database, mostly used by travel agencies etc., that contains data on number and duration of trips to each country is available, making it possible to estimate also the incidence of travel-associated infections. This database has previously been used to study the incidence and risk for Swedish international travellers for malaria, dengue, hepatitis A, campylobacteriosis, salmonellosis and shigellosis [7–13]. In this study we, rather than study one pathogen at a time as had been done in previous studies, we grouped notifiable pathogens by their mode of transmission (also including one group with diseases vaccinated against in the Swedish childhood vaccination programme), since the mode of transmission is often more relevant than pathogen when providing recommendations to international travellers. In addition to notifiable disease we also studied self-reported gastro-intestinal and respiratory infections occurring during international travel.

## Methods

### Objective

To identify countries associated with a high number and/or a high incidence of notifiable or self-reported travel-associated infections for Swedish international travellers in order to provide data to Swedish travel medicine practitioners and public health authorities, allowing them to target relevant advice to persons travelling to these countries.

### Study design

The study was a retrospective ecological study.

### Study population

The study population comprised of Swedes <75 years who had been travelling abroad for at least one day in the five-year period 2009–2013. We chose a five-year study period to limit the stochastic variations. A longer study period would give a less accurate description of the current situation because travel patterns and risk for different infections change over time.

### Data sources

#### The travel database

The travel database (Resedatabasen) is a commercial database that contains data on international travel by Swedish residents 1989–2013 [14]. This data was generated through monthly telephone surveys of a randomized selection of 2,000 Swedish households. To be selected for a telephone interview the household had to have a landline or cell phone number and at least one of the household members had to be younger than 75 years and had to be Swedish speaking. The interviews were undertaken both at daytime and during evenings to reach day-time workers. The first question to a selected household member was if any of the household members had been traveling abroad during the last month. If the answer was yes questions such as age, sex, destination(s) for travel, duration of travel and self-reported gastrointestinal or respiratory infections followed. The answers could be extrapolated to the general Swedish population by applying a weight to each answer. This weight was calculated by the managers of the travel database. Weights were applied each month so that the data would be representative of the Swedish population (according to official statistics) in terms of age, sex and county of residence. For example, if the sample one particular month contained answers from 1.9% of people from a county where 1.64% of the population lives, those answers were given a weight of 0.863 (1.64/1.90 = 0.863)

#### Notifiable infections

Data on notifiable infections was extracted from the Swedish national reporting system for notifiable diseases, which is managed by The Public Health Agency of Sweden. Notifiable diseases are regulated in the Swedish Communicable Disease Act and include 64 pathogens. For some pathogens only a particular disease manifestation (e.g. invasive infections) is notifiable. For other pathogens only isolates with a particular pattern of resistance to antimicrobial drugs are notifiable. Notifications are made in parallel by the clinician diagnosing the patient and the clinical microbiological laboratory that analysed the specimen. The notification from the clinician contains information on age, sex, suspected country of infection and other epidemiological data when appropriate. The two notifications are then merged into a “case” using the unique personal identification number that is given to each Swedish citizen. Refugees and those seeking citizenship are given a temporary number that allows the same possibility to merge clinical and microbiological data into a single “case”.

### Measures to minimize bias

In order to have comparable and relevant data from the two data sources we applied some exclusion criteria to the national database for notifiable infections. Since the purpose of the study was to study the risk of travel-associated infections we did not include infections with pathogens that can cause chronic infection e.g. HIV, chronic hepatitis B and tuberculosis, since it is harder to determine when cases were infected i.e. even if a case was diagnosed after a trip to a particular country it is not certain that the case had been infected during the trip. The variable “suspected country of infection” in the national database on notifiable disease is problematic in a sense that it does not differentiate between infections that occurred during travel and infections that occurred in another country before an individual moved to Sweden and became a Swedish citizen. Thus “suspected country of infection Thailand” could both be a Swedish citizen that was infected during a trip to Thailand, or an immigrant from Thailand who was infected before coming to Sweden. This bias was partly removed by not including chronic infections. A further step that was taken to reduce this bias was to remove all notifications of cases who did not have a permanent Swedish personal identification number. Thus we excluded immigrants who were infected before arrival to Sweden but diagnosed in Sweden upon arrival as they also would be reported with their country of origin as the “Suspected country of infection”, e.g. an immigrant from Iraq who was diagnosed with Chlamydia upon arrival to Sweden would be reported with “Suspected country of infection: Iraq”. To some extent this group was also excluded from the denominator data since the travel database only included Swedish speakers, and newly arrived immigrants are less likely to be Swedish speakers. And since the travel database included only those <75 years we excluded all cases who were older than 74 years old.

### Data analysis

#### Case definitions

For self-reported infections, cases were defined as those who reported having either gastro-intestinal infection or a respiratory illness during international travel between 2009–2013 according to the answers in the travel database.

For notifiable infections, cases were defined as those <75 years of age with a Swedish personal identifier number (excluding those with temporary numbers) and a notifiable infection between 2009–2013 and who, according to the notification, were infected abroad.

We grouped notifiable infections according to their mode of transmission into three groups, we also included one group with notifiable infections vaccinated against in the Swedish childhood vaccination programme.

Food—and waterborne diseases: Campylobacteriosis, salmonellosis, giardiasis, shigellosis, EHEC-infection, cryptosporidiosis, Entamobae histolytica infection, yersinosis, hepatitis A, paratyphoid fever, typhoid fever, hepatitis E, listeriosis and cholera.

Sexually transmitted diseases: Chlamydia, gonorrhea and acute hepatitis B.

Vector-borne diseases: Dengue fever, malaria, West Nile fever, Japanese encephalitis and yellow fever.

Disease vaccinated against in the Swedish childhood vaccination programme: Tetanus, pertussis, measles, mumps, rubella and diphtheria.

#### Denominator calculations

We extrapolated the number of travel days per country to the entire Swedish population by multiplying each answer with the calculated weight. Countries were grouped into continents using the United Nations definition of continents [15]. For the analysis, territories in other continents were considered to be part of the continent where they are situated. E.g. Martinique was considered to be a separate country belonging to Latin America, even though it is a part of France. An exception was the Canary Islands, which we considered to be a part of Spain and Europe.

#### Statistical methods

We calculated the number of infections and incidence per million days of travel for self-reported gastro-intestinal and respiratory infections by multiplying each reported episode with the calculated weight. This was done per continent and per age-group. We also calculated the number of self-reported gastro-intestinal and respiratory infections for each country in the same way.

We chose to present the incidence per million days of travel in order to achieve an incidence between 1–100 for most diseases. Other studies have chosen to present incidence in other ways that are possibly easier to interpret for travellers or travel-medicine practioners e.g. 1 per x number of travellers per month of travel [16]. Since our report is meant to provide data for policy makers we choose to present data as incidence per million days of travel since it can easily be converted in to a different way of presenting risk when necessary.

Since the incidence for destinations to where people travel less often could be more prone to stochastic variations we decided to calculate the incidence for each disease group for the five countries with the highest number of infection instead of calculating the five countries with the highest incidence.

For the five countries with the highest number of self-reported gastro-intestinal and respiratory infections we calculated the incidence per million days of travel.

The number of infections for notifiable infections was also calculated per country and per continent. For the five countries with the highest number of infections per disease group we calculated incidence per million days of travel.

The data was analysed using STATA version 12 (StataCorp. 2011. Stata Statistical Software: Release 12. College Station, TX: StataCorp LP.)

### Ethical approval

The Public Health Agency of Sweden makes use of the data in the national database for notifiable infections as regulated in the Communicable Disease Act. The Travel Database contains anonymized data. This study was undertaken as part of the Public Health Agency of Sweden’s duties. Therefore obtaining ethical approval by an external review board was waived.

## Results

### Travel database

**Fig 1:**
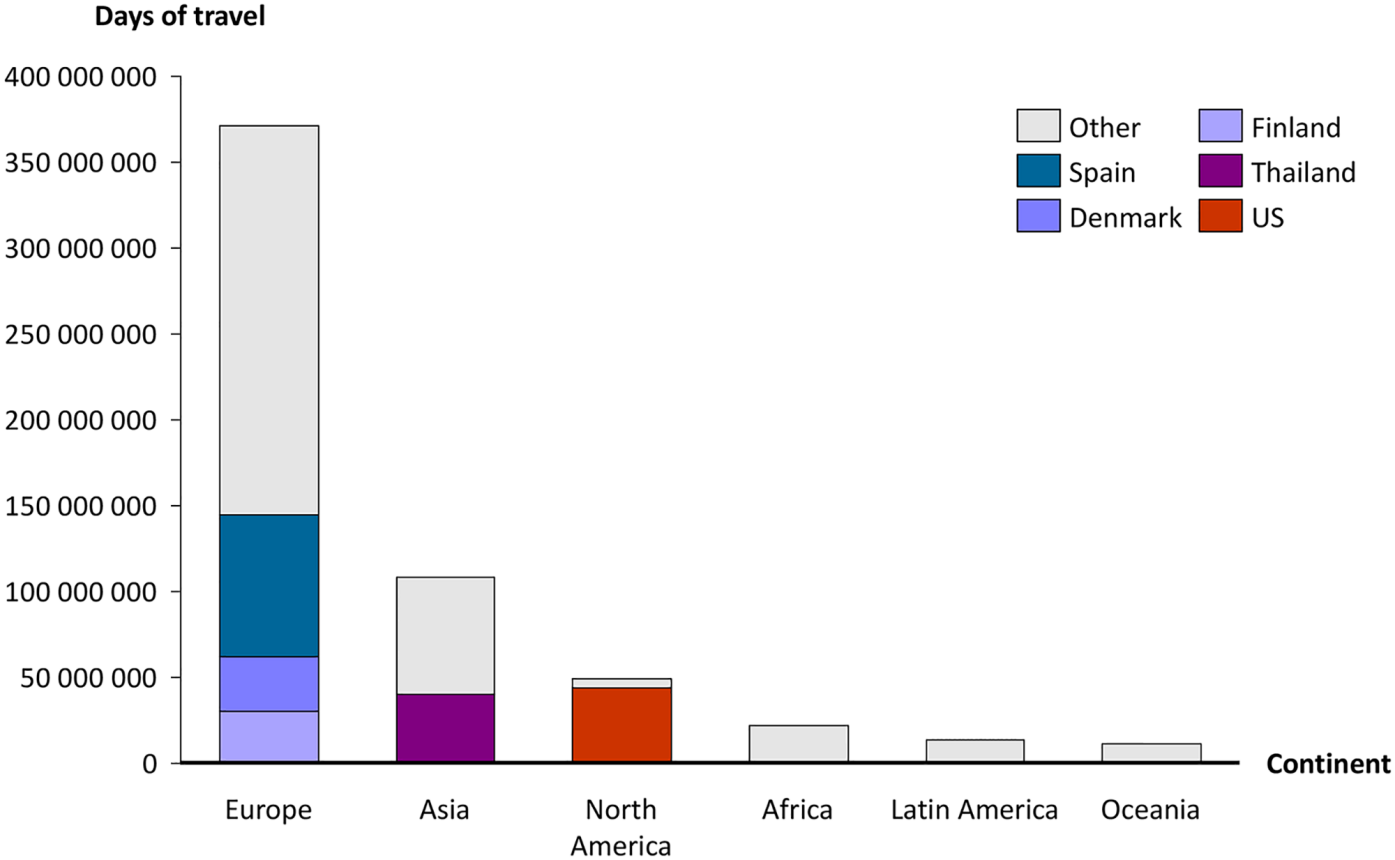
Days of international travel per continent. Coloured bars represent the five countries with highest number of days of travel. International travellers. Sweden 2009–2013.

Between 2009–2013, the travel database contained 18,507 interviews with respondents who had travelled abroad the month before being interviewed. Using the weights we extrapolated the data to 83,646,408 trips for a total of 576,356,972 days of travel for the entire Swedish population during this period. Based on the Swedish population in 2013 (9,644,864) this corresponded to a yearly average of 1.7 trips per person and 11.9 days of travel per person per year. Most days of travel were within Europe followed by Asia (Fig 1). The most popular destination was Spain followed by the US and Thailand.

### Self-reported infections

**Fig 2:**
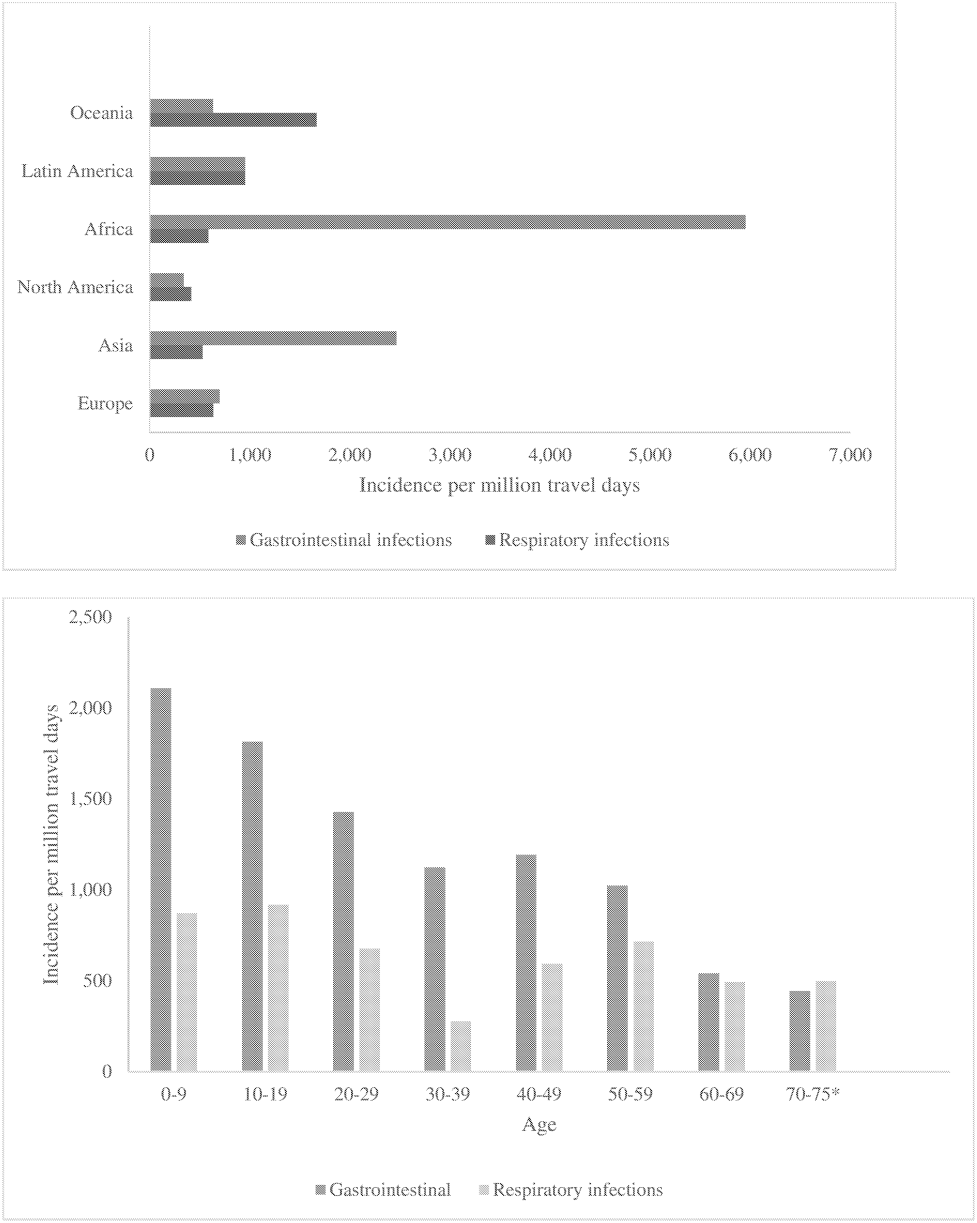
**a**. Incidence of self-reported gastro-intestinal and respiratory infections, per continent. International travellers. Sweden 2009–2013. **b**. Incidence of self-reported gastro-intestinal and respiratory infections per age group. International travellers. Sweden 2009–2013.

The incidence of gastro-intestinal infections was highest for travel to Asia (Fig 2a) and in the 0–9 years age group (Fig 2b). The incidence of respiratory infections was highest for travel to Oceania (Fig 2a) and in the 10–19 years age group (Fig 2b). For gastro-intestinal infections most infections occurred in Thailand but the incidence was highest for Egypt (Table 1). For respiratory infections most infections occurred in Spain but the incidence was higher in Norway.

**Table 1:** Number of self-reported gastro-intestinal and respiratory infections, and incidence per million days of travel, for the five countries with the most infections. International travellers. Sweden 2009–2013.

**Gastro-intestinal infections**			
**Country**	**Travel days**	**Cases**	**Incidence per million days of travel**
Thailand	40,378,716	132,145	3,273
Egypt	6,804,047	82,424	12,114
Spain	82,284,385	78,927	959
Turkey	23,722,180	50,951	2,148
Greece	25,955,789	25,451	981
**Respiratory infections**			
**Country**	**Travel days**	**Cases**	**Incidence per million days of travel**
Spain	82,284,385	59,149	719
Norway	28,700,562	22,705	791
USA	44,280,212	20,635	466
Thailand	40,378,716	20,456	507
Greece	25,955,789	18,662	719

### Notifiable infections

During 2009–2013 there were 47,633 notifications for the diseases we had selected with a country other than Sweden as the suspected country of infection. Of these, we excluded 1,451 since the temporary personal identification number indicated that they referred to newly arrived immigrants, leaving 46,182 notifications for analysis.

Food—and waterborne diseases were the type of notifiable travel-associated infections with highest incidence (61 cases per million days of travel), followed by sexually transmitted diseases (17 cases per million days of travel) (Table 2). Campylobacteriosis was the type of food- and waterborne disease with the most number of cases representing 52% (18,219/35,044) of all food- and waterborne diseases and chlamydia was the type of sexually transmitted disease with the highest number of cases representing 88% (8,770/9,958) of the sexually transmitted diseases (Table 2).

**Table 2:** Number of notifiable infections, and incidence per million days of travel, for travel-associated notifiable diseases, per continent. Returning international travellers. Sweden 2009–2013.

Continent	Europe		Asia		North America		Africa		Latin America		Oceania		Total	
Days of travel	371,384,907		108,315,711		49,412,296		22,026,333		13,626,739		11,590,986		576,356,972	
	Cases	Incidence	Cases	Incidence	Cases	Incidence	Cases	Incidence	Cases	Incidence	Cases	Incidence	Cases	Incidence
**Food- and waterborne diseases**	**9,173**	**25**	**18,046**	**167**	**80**	**2**	**6,483**	**294**	**1,204**	**88**	**58**	**5**	**35,044**	**61**
Campylobacteriosis	6,553	18	9,369	86	45	1	1,746	79	476	35	30	3	18,219	32
Salmonellosis	1,767	5	5,709	53	10	0.2	2,010	91	241	18	18	2	9,755	17
Giardiasis	287	1	1,535	14	17	0.3	1,291	59	275	20	9	1	3,414	6
Shigellosis	66	0	545	5	0	0	835	38	105	8	0	0	1,551	3
EHEC-infection	191	1	260	2	2	0.04	237	11	37	3	0	0	727	1
Cryptosporidiosis	122	0.3	162	1	6	0.1	106	5	21	2	1	0.1	418	1
Entamobea histolytica	19	0.1	167	2	0	0	151	7	18	1	0	0	355	1
Yersinosis	125	0.3	60	1	0	0	28	1	19	1	0	0	232	0.4
Hepatitis A	34	0.1	104	1	0	0	73	3	5	0.4	0	0	216	0.4
Paratyphoid fever	3	18	53	0.5	0	0	2	0.1	4	0.3	0	3	62	0.1
Typhoid fever	0	0	50	0.5	0	0	4	0.2	0	0	0	0	54	0.1
Hepatitis E	1	0.003	26	0.2	0	0	0	0	2	0.1	0	0	29	0.1
Listeriosis	5	0.01	2	0.02	0	0	0	0	1	0.1	0	0	8	0.01
Cholera	0	0	4	0.04	0	0	0	0	0	0	0	0	4	0.01
**Sexual-transmitted diseases**	**5,083**	**14**	**3,167**	**29**	**420**	**8**	**495**	**22**	**395**	**29**	**398**	**34**	**9,958**	**17**
Chlamydia	4,640	12	2,590	24	382	8	417	19	350	26	391	34	8,770	15
Gonorrhea	418	1	513	5	35	1	63	3	41	3	7	1	1,077	2
Acute Hepatitis B	25	0.1	64	1	3	0.1	15	1	4	0.3	0	0	111	0.2
**Mosquito-borne diseases**	**9**	**0**	**563**	**5**	**0**	**0**	**290**	**13**	**57**	**4**	**1**	**0.1**	**920**	**2**
Dengue fever	7	0.02	532	5	0	0	8	0.4	54	4	1	0.1	602	1
Malaria	0	0	30	0.3	0	0	282	13	3	0.2	0	0	315	1
West Nile fever	2	0.01	0	0	0	0	0	0	0	0	0	0	2	0.003
Japanese encefalitis	0	0	1	0.01	0	0	0	0	0	0	0	0	1	0.002
Yellow fever	0	0	0	0	0	0	0	0	0	0	0	0	0	0
**Disease vaccinated against in the Swedish childhood vaccination programme**	**48**	**0.1**	**38**	**0.4**	**3**	**0.1**	**9**	**0.4**	**4**	**0.3**	**1**	**0.1**	**103**	**0.2**
Pertussis	19	0.1	9	0.1	2	0.04	2	0.1	3	0.2	1	0.1	36	0.1
Measeles	15	0.2	14	0.1	0	0	2	0.1	0	0	0	0	31	0.1
Mumps	12	0.03	11	0.1	1	0.02	3	0.1	1	0.1	0	0	28	0.05
Rubella	2	0.01	3	0.03	0	0	0	0	0	0	0	0	5	0.01
Diphteria	0	0	1	0.01	0	0	2	0.1	0	0	0	0	3	0.01
Tetanus	0	0	0	0	0	0	0	0	0	0	0	0	0	0

The destination with the highest number of food- and waterborne diseases and sexually transmitted diseases was Thailand, with 7,697 and 1,388 cases respectively (Table 2). India and Egypt had a higher incidence of food- and waterborne diseases (388 and 248 cases per million days of travel compared to Thailand with 191) while Thailand was the country with the highest incidence of sexually transmitted diseases (34 per million days of travel). (Table 3).

**Table 3:** Number of notifiable infections, and incidence per million days of travel, for the five countries with the most infections per disease group. Returning international travellers. Sweden 2009–2013.

**Food- and waterborne diseases**				**Sexual transmitted diseases**			
**Country**	**Travel days**	**Cases**	**Incidence per million days of travel**	**Country**	**Travel days**	**Cases**	**Incidence per million days of travel**
Thailand	40,378,716	7,697	191	Thailand	40,378,716	1,388	34
Turkey	23,722,180	3,681	155	Spain	82,284,385	967	12
Spain	82,284,385	2,928	36	Norway	28,700,562	737	26
India	6,560,155	2,546	388	Turkey	23,722,180	544	23
Egypt	6,804,047	1,689	248	Greece	25,955,789	465	18
**Vector-borne diseases**				**Diseases vaccinated against in the Swedish childhood vaccination programme**			
**Country**	**Travel days**	**Cases**	**Incidence per million days of travel**	**Country**	**Travel days**	**Cases**	**Incidence per million days of travel**
Thailand	40,378,716	358	9	France	21,465,557	8	0.4
Indonesia	1,968,274	65	33	Thailand	40,378,716	7	0.2
India	6,560,155	42	6	Spain	82,284,385	7	0.1
Uganda	409,758	41	100	India	6,560,155	5	0.8
Nigeria	0	34	0	Great Britain	26,228,580	5	0.2

## Discussion

We used the unique situation in Sweden where both numerator data on self-reported and notifiable infections as well as denominatordata on number and lengths or trips are available to study travel-associated infections among Swedish international travellers. This made it possible for us to calculate both the number of notifiable infections and self-reported gastro- and respiratory infections that occurred among Swedish international travellers as well as the incidence per country and thus describe both the burden in terms of number of infections and the risk in terms of incidence per country and million days of travel.

We identified several interesting associations concerning self-reported infections:

Self-reported gastro-intestinal infections were almost twice as common as self-reported respiratory infections, which is in line with findings in previous studies from Sweden, other countries in Europe and the US [2, 5, 17–21]. Self-reported food- and waterborne infections had the highest incidence during trips to Africa. This was also the case for notifiable food- and waterborne infections. That gastro-intestinal infections are common during trips to Africa has previously been demonstrated [2, 21]. This could be due to poor hygienic conditions in parts of Africa. The highest number of self-reported gastro-intestinal infections occurred in Thailand but the incidence was four times higher in Egypt. The number of self-reported gastro-intestinal infections during travel to Thailand was 17 times higher than the number of notifiable food- and waterborne infection among returning international travellers who had visited Thailand. This probably reflects that many infections are self-limiting and does not require health care upon return to Sweden and are therefore never diagnosed and notified. In addition, previous studies have shown that many of the gastro-intestinal infections that occur during travel are norovirus and *E coli* [22] and they are not notifiable in Sweden.

Self-reported respiratory infections had the highest incidence during trips to Oceania, followed by Latin America and Africa. A reason for this could be that travel to these destinations takes longer time and long air trips could be a risk factor for respiratory illness. Either by respiratory infections such as influenza can spread in the cabin of an aircraft which has previously been shown [23, 24]. A longer flight would then mean a longer time for a possible exposure to airborne pathogens in the cabin. Or it could be because mucosal membranes are damaged by the dry air in the cabin and the risk for respiratory infections is elevated after the air trip. Another likely explanation could be that the seasons are reversed in the Southern hemisphere and influenza and other airborne viruses are circulating.

We also made interesting observations on the notifiable infections:

Campylobacteriosis was the type of notifiable travel-associated food- and waterborne disease with the highest number of cases. Most food- and waterborne infections were associated with travel to Asia followed by travel to Europe. In Europe campylobacteriosis is the most common cause of diarrheal disease [25]. In the US it is the second most common after salmonellosis [26]. According to the WHO campylobacteriosis is one of the most frequently occurring agents of bacterial gastroenteritis [27]. A previous study on European travellers seeking health care at travel medicine clinics Giardia was more common than Campylobacter in those with acute diarrhoea [5]. This difference could perhaps be explained by the difference in the population studied. Among returning travellers with acute diarrhoea those with giardiosis might be more likely to seek travel medicine care than those with campylobacteriosis. Thailand was the country with the highest number of notifiable food- and waterborne diseases.

Chlamydia was the type of notifiable travel-associated sexually transmitted diseases with the highest number of cases. Most notifiable sexually transmitted diseases occurred during trips in Europe. But Thailand was the country associated with the highest number of notifiable sexually transmitted diseases. Norway was the country after Thailand and Spain where most notifiable sexually-transmitted infections occurred. This could possibly be explained by the many Swedish youths that go to Norway for short-term work and that youths are at higher risk for sexually-transmitted diseases. A previous study on Swedish youths have shown that age between 18–24 years, one month or more of travel and heavy episodic drinking are risk factors for sexual risk-taking abroad [28].

Most notifiable travel-associated vector-borne diseases occurred among travellers to Asia (where dengue fever was more common than malaria) although the incidence per million travel-days was higher for Africa (where malaria were more common than dengue fever). Thailand was the country associated with the highest number of notifiable vector-borne diseases (with mostly dengue fever). But Uganda and Indonesia had higher incidence per million travel-days (with malaria in Uganda and mostly dengue fever in Indonesia). Overall there were twice as many cases of dengue fever compared to cases of malaria. This is different from the previously mentioned study on returning Europeans travellers seeking travel medicine care [5]. In that study malaria was more common than dengue fever perhaps reflecting that those with malaria are more likely to seek specialized travel medicine care than those with dengue fever.

Most infections vaccinated against in the Swedish childhood vaccination programme were associated with travel within Europe. It was the least common type of notifiable travel-associated infections studied. This could reflect the high uptake of childhood vaccinations in the Swedish population [29]. These findings were similar to those in a study from Finland where diseases that could be prevented by vaccinations were rare among international travellers [21].

### Limitations

A general limitation was that, apart from self-reported gastro- and respiratory infections, only notifiable diseases were included. Thus this is not a complete survey of infections of all types during travel.

One had to be a Swedish speaker to be included in the travel database. This could have led to a bias in that Swedish citizens who are not Swedish speakers might have a different travel pattern than those who are Swedish speakers. They are probably more likely to be born outside of Sweden and, when they travel, more likely to visit relatives and interact with the local population and therefore of higher risk of some infections. They would be captured by the surveillance for notifiable infections but not in the travel database and the estimated incidence for the countries that they travel to would be overestimated.

For vector-borne infections we found 34 cases reported being infected in Nigeria but no travels-days reported for Nigeria. This might reflect a weakness in the precision of the data on number of travels to countries where people travel to less often. The number of interviews per month may not be enough to capture travel to rare destinations. This is however unlikely to affect the estimates for countries to which Swedes travel more frequently. A different explanation could be that those who travel to Nigeria are less likely to be Swedish speakers and/or have a Swedish telephone number and therefore would not end up being interviewed. An additional limitation is that the findings from this study might not me generalizable for other populations than the Swedish population since travel patterns and risk taking behaviour might be different for other populations.

### Conclusion

In conclusion we have identified travellers to Thailand as a particular group that could be targeted by preventive measures that minimize the number of travel-associated infections since Thailand was the country associated with highest number of notifiable food- and waterborne infections, sexually transmitted infections and vector-borne infections. We also found that Europe was the continent where the most notifiable sexually transmitted infections and infections with vaccinated against in the Swedish childhood vaccination programme occurred as well as the continent with the most notifiable food- and waterborne diseases after Asia.

### Recommendation

We recommend to target public health interventions and travel medicine advice especially to those travelling to Thailand, but not disregard the risks of travel-related infections that occur during travel within Europe.
